# COVID-19 vaccine equity and the right to health for displaced Venezuelans in Latin America

**DOI:** 10.1371/journal.pgph.0001275

**Published:** 2023-03-01

**Authors:** David C. Hill, Zafiro Andrade-Romo, Karla Solari, Ellithia Adams, Lisa Forman, Daniel Grace, Alfonso Silva-Santisteban, Amaya Perez-Brumer

**Affiliations:** 1 Social and Behavioural Health Sciences Division, Dalla Lana School of Public Health, The University of Toronto, Toronto, Ontario, Canada; 2 Unit of Health, Sexuality, and Human Development, Universidad Peruana Cayetano Heredia, Lima, Peru; University of Washington Bothell, UNITED STATES

## Abstract

Given the magnitude of Venezuelan displacement in Latin America, there is a need to assess how migrants were, and will continue to be, addressed in COVID-19 vaccination policies. To explore migration status as a dimension of vaccine equity in Latin America and in relation to international human rights, we assessed national vaccination plans, peer-reviewed, and gray literature published between January 2020 and June 2021. Three key rights-related concerns were found to restrict the health rights of migrants in the region: 1) lack of prioritization of migrants in vaccine distribution; 2) onerous documentation requirements to be eligible for COVID-19 vaccination; and (3) how pervasive anti-migrant discrimination limited equitable health care access. While international human rights law prohibits against discrimination based on migration status, few countries analyzed realized their obligations to provide equal access to COVID-19 vaccines to non-citizens, including displaced Venezuelans. Especially for migrants and displaced people, effective and sustainable vaccination strategies for COVID-19 and future pandemics in Latin America must be guided not only by epidemiological risk but also seek to align with human rights obligations. To achieve this, States must also take special measures to facilitate vaccine access for communities facing systemic discrimination, exclusion, and marginalization.

## Introduction

In January 2021, Dr. Tedros Adhanom Ghebreyesus, Director-General of the World Health Organization (WHO), described the hoarding of COVID-19 vaccines by wealthy nations as a “catastrophic moral failure” that would “only prolong the pandemic, the restrictions needed to contain it, and human and economic suffering” [1]. Yet despite repeated calls for an equitable, globally coordinated approach to COVID-19 vaccination, vaccine apartheid has been unaddressed; as of March 2021, 78% of the 447 million doses were deployed in just ten countries [2]. By August 2021, over half of all people were fully vaccinated in Canada, the United States, the United Kingdom, and other Western countries, compared with just 11% in Latin America and the Caribbean [3–6]. These gross disparities are poised to deepen as third and fourth doses are increasingly recommended by governments and public health officials across North America and Europe [7]. Within countries already at the end of the line for doses, marginalized communities face additional barriers to access COVID-19 vaccines [8]. On both a global and a national scale, policymakers are grappling with how to prioritize COVID-19 vaccination to slow pandemic spread and protect the most vulnerable, including migrants.

Greater attention to migration status as a determinant of COVID-19 vaccine equity is urgently needed. Irregular migration status often intersects with experiences of “racism, patriarchy, economic disadvantages, and other discriminatory systems…to create layers of inequality” [9]. In 2000 the Committee on Economic, Cultural, and Social Rights (CESR) affirmed that “States are under the obligation to respect the right to health by, inter alia, refraining from denying or limiting equal access for all persons, including prisoners or detainees, minorities, asylum seekers and illegal immigrants, to preventive, curative and palliative health services” [10]. Yet, some restrictions of rights based on irregular migration status may be justified; for example, the International Convention of Migrant Workers (ICMW) reduces the right to health for migrants in an irregular situation to “medical care that is urgently required for the preservation of life or the avoidance of irreparable harm to their health” [11]. The CESR further specified that irregular migrants are to be considered ‘specifically vulnerable’ people, recalling the New York Declaration for Refugees and Migrants which “reaffirmed the human rights of all refugees and migrants, regardless of status” [12]. In March 2020, the United Nations further stipulated that when a vaccine for COVID-19 became available, it should be provided “without discrimination”, that resource scarcities “should never be used as a justification to discriminate against certain groups of patients”, and that a human rights approach is effective in preventing “major public health threats” [14].

Yet, COVID-19 vaccine access has repeatedly been denied to migrants, particularly those with irregular migration status, referring to migrants who entered a country without authorization or required documentation and often lack access to basic rights, including health care [13, 14]. By May 2022, out of 180 countries evaluated globally by the United Nations International Organization for Migration, 39 countries excluded migrants with an irregular status from COVID-19 vaccination plans, underscoring the precarity of irregular migrants produced in part through state-supported exclusion from social protections [15]. Exclusion from COVID-19 vaccination efforts based on migration status raises important concerns surrounding State protections and the health rights of migrants in an irregular status amid public health emergencies.

By August 2022, more than 6.8 million Venezuelans fled the country due to political, economic, and social turmoil, making it the largest international displacement in Latin America and the Caribbean in contemporary history [16]. Most Venezuelans remain in South America, placing severe socioeconomic strains on countries such as Argentina, Brazil, Chile, Colombia, Ecuador, and Peru, which collectively host over 5.1 million Venezuelans [17]. While both regular and irregular migrants face stigmatization, discrimination, and health care access barriers, lack of documentation can make these issues particularly salient for irregular migrants. For many countries, government-issued identification documents (IDs) are required to access COVID-19 vaccinations. ID requirements coupled with heightened xenophobia and ongoing human rights violations may negatively impact how Venezuelan migrants are included in host country vaccination policies [18].

While the Latin American region has been significantly impacted by Venezuelan mass migration, limited research has assessed the sociopolitical climates shaping COVID-19 vaccine access for displaced Venezuelans. Thus, there is an urgent need to understand how rising numbers of displaced Venezuelans will be addressed in country-level COVID-19 vaccination policies. Addressing this gap, between April and June 2021, we examined the bioethical and human rights dimensions of country-level COVID-19 vaccination policies for migrants. This research builds on existing work linking health inequities to societal and structural-level forces, such as racism, capitalism, power, and politics [19–21]. In doing so, we theorize health as inextricably linked to not just individual-level decisions or community factors, but also broader societal forces. While results are inherently limited by the timeframe of our search, we aim to better understand migration status (i.e. regular and irregular) as a dimension of vaccine equity through country-level vaccination plans and in relation to international human rights obligations and the “WHO SAGE values framework for the allocation and prioritization of COVID-19 vaccines”, which provides guidelines to promote equity within global and national prioritization of vaccination.

## COVID-19 vaccination facilities and equity frameworks

In recognition of underlining inequities pervasive in emergency vaccine administration, the “WHO SAGE values framework for the allocation and prioritization of COVID-19 vaccines” (hereafter referred to as the WHO/SAGE framework) was developed “for COVID-19 vaccines to contribute significantly to the equitable protection and promotion of human well-being among all people of the world” [22]. The WHO/SAGE framework is guided by a bioethical approach emphasizing that social vulnerabilities (including citizenship status) should be considered when prioritizing COVID-19 vaccination. However, as Sekalala and colleagues note, while the WHO/SAGE framework mentions human rights principles and references State obligations to promote ‘global equity’, it falls short of human rights obligations to hold States accountable to ensure equity in access to vaccines [23].

Second, the need for international collaboration led to the creation of COVID-19 Vaccines Global Access (COVAX) to support vaccine research and development and promote global equity in vaccine distribution [24]. COVAX contains a small “humanitarian buffer” (around 5 percent of doses), which includes doses for migrants and refugees who otherwise do not have access through national vaccination campaigns. Yet, the number of doses available through COVAX is wholly inadequate given the magnitude of the Venezuelan migration crisis and other migration crises [25]. COVAX does not specify how national prioritization of vaccination should proceed, leaving governments to define priority groups for COVID-19 vaccination.

Existing COVID-19 vaccination facilities and equity frameworks may not adequately account for the unique vulnerabilities facing migrants amid COVID-19. For example, the WHO/SAGE framework, which is meant to guide State actors, lacks accountability in upholding the health rights of non-citizens. Scholars have thus argued that COVID-19 vaccination frameworks can be strengthened by aligning with international human rights obligations that define health rights; this can create legally enforceable frameworks that can be used to hold States accountable for protecting rights, particularly amid emergencies such as COVID-19 [23]. Indeed, international human rights treaties (including the International Covenant on Civil and Political Rights and the International Covenant on Economic, Social, and Cultural Rights) are ratified and legally binding in most countries globally. While the ICMW only guarantees access to emergency lifesaving emergency medical care, the United Nations CESR has emphasized that access to COVID-19 vaccines is “an essential component of the right of everyone to the enjoyment of the highest attainable standard of physical and mental health and the right of everyone to enjoy the benefits of scientific progress and its applications” [26]. The Committee also underscores the obligation of States “as a matter of priority and to the maximum of their available resources, to guarantee all persons access to vaccines against COVID-19, without any discrimination” [26]. The integration of human rights into existing vaccination frameworks would transform ethical imperatives into obligations and rights, which can be mobilized in legal and political strategies by civil society and social actors to hold States accountable for ensuring health rights are fulfilled [23].

## Methods

### Ethics statement

The research presented in this manuscript is based on a scoping review of published and/or publicly reported literature and thus did not require institutional ethical approval.

Data are derived from a scoping review conducted between April and June 2021 evaluating the inclusion of Venezuelan migrants in COVID-19 vaccination policies in Latin America. Complete scoping review methods have been previously published [27]. The original scoping review broadly assessed COVID-19 vaccine access for displaced Venezuelans, while this secondary analysis explored the human rights dimensions of COVID-19 vaccination for displaced Venezuelans. This analysis sought to better understand the human rights dimensions of vaccine access, advancing understandings of initial country-level approaches to COVID-19 vaccination and the complex bioethical and human rights dimensions of vaccine equity amid Venezuelan mass migration and COVID-19 in Latin America. In-depth review consisted of full-text review of 13 peer-reviewed articles, analysis of country-level (and at times provincial or municipal) vaccination plans, and gray literature searches across websites for non-governmental organizations, multilateral agencies, conference proceedings, and news media, producing a rich dataset for analysis. Vaccination plans and gray literature were examined alongside international human rights obligations and the WHO/SAGE framework to explore how human rights were framed with regards to COVID-19 vaccine access for Venezuelan migrants.

Four team members contributed to data extraction with at least one full text reviewer per data source. A search of peer-reviewed literature published between January 2020 and June 2021 yielded 142 results and 13 articles included in analysis; gray literature screening included 74 materials for full text review and 37 included in analysis, including publications and reports by nongovernmental organizations, multilateral agencies, and news media; and country-level documents were reviewed for 18 countries across Latin America and the Caribbean, with in-depth analysis for the six countries hosting most Venezuelans (Argentina, Brazil, Chile, Colombia, Ecuador, and Peru). Two team members reviewed Ministry of Health websites, policies, documents, and media related to COVID-19 vaccination plans. Analysis drew on a rights-based approach to identify relevant human rights literature related to COVID-19 vaccination of displaced Venezuelans evidenced across the peer-reviewed and gray literature.

Data were collected in English, Spanish, and Portuguese through a variety of online platforms for screening and data extraction (Covidence, Google Forms, Zotero, and Excel). Data extraction was divided by data source (peer-reviewed, country-level government policies, gray literature) with at least one full-text reviewer and data extractor per publication. Summative content analysis guided this analytic process to assess both what is explicit as well as implicit with regards to mechanisms for COVID-19 vaccine access based on migration status [28, 29]. Research team coded sections of the documents into overarching interpretative themes and subthemes related to how the human rights of migrants were explicitly described or implied in the sources and, in some cases, excluded from vaccination strategies. Codes were used to generate key themes across extracted documents and yielded summaries relevant to bioethics, human rights, law, policy, and institutional practices for migrants. Emergent themes were discussed across the full team (6 members) who met twice weekly throughout the six-week study period to review extraction procedures and to compare, discuss, and interpret data.

## Results

**Fig 1:**
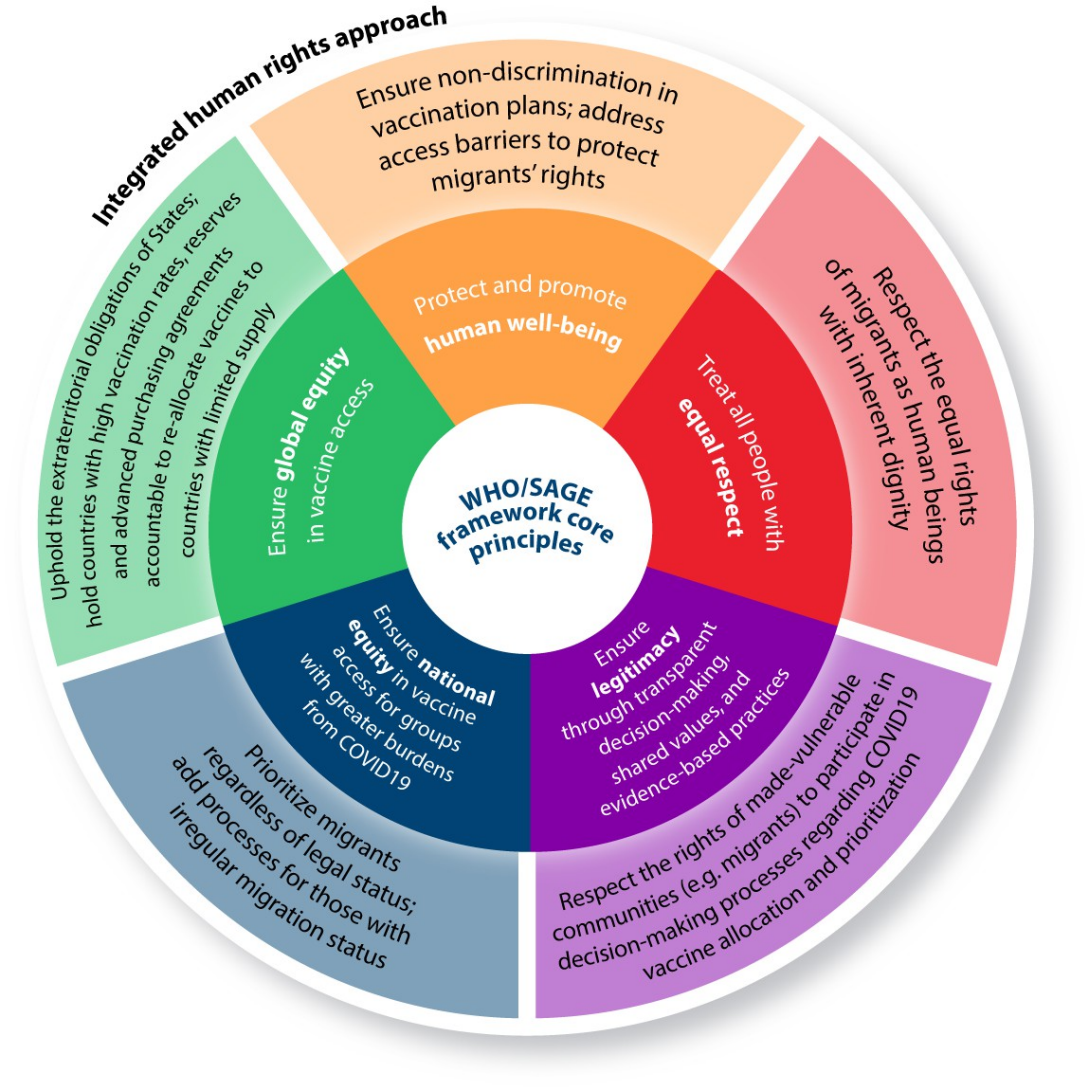
Integrating human rights into a modified WHO/SAGE framework to address the unmet COVID-19 needs of migrants. Inner circle: Summary of select principles in the WHO/SAGE framework. Outer circle: Integrating human rights with WHO/SAGE to promote equitable access to COVID-19 vaccines for migrants.

Across the analysis, three key rights-related concerns were found to restrict the right to health for migrants in Latin America amid COVID-19: 1) lack of prioritization of migrants in vaccine distribution; 2) onerous documentation requirements to be eligible for COVID-19 vaccination; and 3) how pervasive anti-migrant discrimination limited equitable health care access. A detailed analysis of country-level vaccination plans, phases of vaccination, and ID requirements for vaccination is presented in Table 1, and Fig 1, in the Discussion, provides recommendations based on results for integrating human rights into existing COVID-19 vaccination facilities and frameworks (i.e. WHO SAGE/values).

**Table 1:** Phases of COVID-19 vaccination, ID requirements, and inclusion of Venezuelan migrants in six host countries as of June 2021.

Country	Phases of Vaccination	ID requirements	(How) are migrants included in vaccination plan?
Argentina^a^ [37]	Phase 1: Adults over 70 years and adults in long-term care residences Phase 2: Adults over 60 years Phase 3: Military personnel, armed forces, and prison staff Phase 4: Adults 18–59 years in “high risk” groups (diabetes, obesity, cardiovascular disease, cancer, etc.) Phase 5: Teachers and school staff Phase 6: Other priority populations to be defined by jurisdictions, **including based on social vulnerability such as migration status**^b^	No ID required for vaccination	Phase 6 prioritizes vaccination based on social vulnerability, including migration status.
Brazil [38]	Priority groups 1–2: Long-term care residents Group 3: Indigenous people on Indigenous lands Group 4: Health workers Groups 5–8: Adults 75 years and older Groups 9–10: Indigenous communities (Ribeirinhas and Quilombola) Groups 11–13: Adults 65–74 years Groups 14–15: Adults with co-morbidities or disabilities Group 16: People experiencing homelessness Groups 17–18: Prison inmates and staff Groups 19–20: Teachers Groups 21–22: Armed forces and first responders Groups 23–29: Industry workers (public transportation, truck drivers, construction, etc.) Group 30: General population 18 years and older	“Cadastro de Pessoas Físicas” [Natural Persons Register] or “Cartão Nacional de Saúde” [National Health Card] required for vaccination	Migrants are not explicitly mentioned in vaccination plans.
Chile [39]	Phase 1: a) Clinical and administrative health personnel, including students in direct contact with patients; b) People residing in long-stay centers, mental health institutions, adults over 70 years c) Government employees, armed forces, first responders d) Adults 60–69 years e) Adults 18–59 years old with comorbidities or with severe disabilities f) Firefighters, personnel in essential services, such as electricity, water, gas, power generation, fuel, telecommunications, waste collection, landfills, foreign trade, public transportation, teachers and childcare providers; prison inmates and staff; Phase 2: General population 18 years and older	ID required for vaccination (e.g., ID document from their place of origin, passport from place of origin, health card). Migrants can self-report to the police to obtain documentation granting access to vaccines.	Migrants are not explicitly mentioned in vaccination plans.
Colombia [40, 41]	Step 1: Adults over 80 years, health care staff working with COVID-19 patients Step 2: Adults 60–79, all other health workers, medical students, traditional healers Step 3: Adults 50–59, people 16–59 with comorbidities, teachers and educational staff, military personnel, police, Indigenous guard Step 4: Adults 40–49, populations at risk of outbreaks (inmates and prison staff, firefighters, first responders, etc.) Step 5: General population 16 years and older	Resident migrants can access vaccines through the *Sistema General de Seguridad Social en Salud*. Irregular migrants must be approved for temporary protection status.	Vaccination plan includes regular migrants but describes the vaccination of irregular migrants as an “ongoing challenge”.
Ecuador [42]	Phase 1: "We save lives": Adults over 65 years, Adults 50–64 with comorbidities or disabilities, health workers, people who live/work in high-risk settings Phase 2: "We take care": Adults 50–64, people aged 16–49 with co-morbidities or disabilities, people who work in strategic sectors Phase 3: "Less contagion": People aged 16–49 years, **mobile populations including irregular migrants** Phase 4: "We reactivate": all those previously unvaccinated	ID is not required. Resident migrants must complete an electronic form for vaccination clinic referral based on place of residence and level of risk. Migrants that are part of other priority groups (based on age, occupation, etc.) can attend mass vaccination clinics without pre-registering.	Phase 3 targets mobile populations including irregular migrants.
Peru [43]	Phase 1: Health care workers and hospital/clinic staff Phase 2: Adults over 60 years, adults with comorbidities, Indigenous communities, prisoners, and correctional staff Phase 3: Adults 18–59 years	Those without Peruvian ID or regular migration status are instructed to register with the National Registry of Identification and Civil Status, which accepts ID documents from country of origin.	Plan states that vaccination will proceed according to priority group regardless of migration status.

^a^ Vaccination plans vary by province; Buenos Aires was selected as a representative case.

^b^ Bolded text indicates where migrants are named as priority groups for vaccination

### Lack of prioritization of migrants in vaccine distribution

Of the six countries reviewed in-depth, only Argentina and Ecuador identified migrants as priority groups for vaccination. In Argentina, migrants were prioritized after adults 60 and over, adults with comorbidities, military personnel, armed forces, prison staff, teachers, and school staff [30]. Similarly, in Ecuador, migrants were listed as a priority group for Phase 3 of the vaccination plan. While vaccination plans in both Argentina and Ecuador clarified that migrants may be eligible alongside citizens if they were part of other priority groups, these were defined by epidemiological risk factors with limited attention to structural factors such as overcrowded housing, precarious employment, and migration status. The first two phases of Ecuador’s vaccination plan, “we save lives” and “we take care”, prioritized adults 50 years and older, adults with comorbidities, and workers in strategic sectors [31]. Phase 3, which explicitly included migrants, was referred to as “limit contagion”, reinforcing stereotypes of migrants as disease vectors. Even when migrants were explicitly named as priority groups for vaccination, this was framed in terms of reducing risk for citizens.

Meanwhile, review of the gray literature revealed a rapidly shifting policy landscape that at times explicitly excluded migrants from vaccination efforts. In Chile, although the Constitution recognizes migrants’ right to health, the Minister of Foreign Affairs stated in February 2021 that irregular migrants would not be vaccinated against COVID-19 [32]. The decision was criticized by the Chilean Medical College, who argued that restricting COVID-19 vaccination based on migration status would disproportionately affect the most vulnerable [33]. In response, the Chilean government updated the policy to only restrict tourists from COVID-19 vaccination [34]. Similarly, in December 2020, Colombia sought to exclude irregular migrants from vaccination plans, including an estimated one million displaced Venezuelans with irregular migratory status [35]. The announcement triggered international outrage. To address the human rights and socioeconomic integration of displaced Venezuelans, in February 2021 President Duque announced a Temporary Protection Status (TPS) program [36]. Migrants eligible for TPS could formalize their status, gain employment legally, and access health care, including vaccination [35]. The discrimination evidenced in statements from government officials clearly juxtaposed human rights approaches that prohibit denying or limiting access to COVID-19 vaccines based on nationality or migration status.

While migrants may be eligible for vaccination based on age or occupation, country-level vaccination plans were often unclear if, how, and when non-citizens, particularly those without IDs, can access vaccines. Table 1 illustrates how reviewed countries allocated their limited COVID-19 vaccines to “at-risk” groups according to age, occupation, and co-morbidities, with limited information on how migrants and non-citizens can access COVID-19 vaccinations within these classifications.

In contrast with Argentina and Ecuador, which explicitly name migrants as priority groups, plans in Brazil, Chile, Colombia, and Peru were more ambiguous regarding access for migrants. Chile and Colombia defined priority groups predominantly by risk of morbidity and mortality (age and co-morbidities), as well as by occupation (health care and other “essential” workers). Yet, while migrants may be eligible based on age, occupation, or comorbidities, vaccination plans were often unclear regarding when and how migrants not eligible based on individual-level risk could access vaccines. Colombia’s plan indicated that while regular migrants could access vaccines alongside citizens, vaccination of irregular migrants was described as an “ongoing challenge” [40]. Some country-level approaches considered group vulnerability when, for instance, prioritizing vaccination in mental health institutions (Chile) and inmates and prison staff (Argentina, Brazil, Colombia, Chile, and Peru).

Findings from peer-reviewed and gray literature reveal how unstable housing, precarious employment, discrimination, and health care access barriers represent intersectional structural-level factors that constrain access to social protections for made-vulnerable communities. In Brazil and Peru, COVID-19 vaccination plans addressed intersectional and structural factors that place Indigenous communities at increased vulnerability. For example, Brazil’s national plan notes collective ways of living coupled with long distances from health centers as factors that place Indigenous Quilombola and Ribeirinhas communities at increased vulnerability to COVID-19, and thus prioritizes these communities for COVID-19 vaccination [38]. Brazil’s prioritization of Indigenous Quilombola and Ribeirinhas communities demonstrates how structural level factors can be considered alongside individual-level risk factors in the allocation and prioritization of vaccines. Yet despite crowded living conditions, health care access barriers, language barriers, and precarious employment, which increase vulnerability to COVID-19 for many displaced Venezuelans in Brazil, migrants as a specific group are not prioritized within Brazil’s vaccination plan [44].

### Onerous documentation requirements to be eligible for COVID-19 vaccination

While some vaccination plans included flexibility surrounding ID requirements for non-citizens or the acceptance of expired IDs, documentation requirements were noted as a recurrent vaccination barrier, particularly for migrants with irregular status [45]. As of March 2021, over 65 percent of Venezuelans in Colombia reported a lack of documentation as a health care access barrier [16]. In Colombia, while regular migrants can access vaccines with a residency permit, irregular migrants must apply for Temporary Protection Status (TPS). Yet, TPS applications can take months to process, further constraining or delaying COVID-19 vaccination [35]. Additional TPS eligibility criteria excluded migrants that arrived after January 31^st^, 2021 and those with a criminal record [16]. Zard et al. documented how relying on regularization processes as a route to vaccination delayed and constrained COVID-19 vaccine access for migrants and refugees [8]. To access vaccines in Colombia, Chile, and Brazil, displaced Venezuelans needed a passport, proof of legal status, or proof of enrolment in health care systems [38, 40]. Registration for COVID-19 vaccination also often required booking an appointment online and uploading documentation, which the International Organization for Migration (IOM) has identified as a barrier for migrants who may lack computer access or internet connectivity [45]. Several support mechanisms were identified in the gray literature to address some of the structural vaccination barriers for displaced Venezuelans, such as United Nations agencies in Brazil assisting with regularization applications, and temporary, transitory, or precarious residency permits being automatically extended in Argentina [46]. These mechanisms sought to facilitate the regularization of migration status for the millions of displaced Venezuelans with irregular migration status in Brazil and Argentina, an important step towards access to social protections such as health care and vaccines.

### How did pervasive anti-migrant discrimination limit equitable health care access?

Another crucial COVID-19 vaccination barrier evidenced across the peer-reviewed and gray literature was migrants’ distrust in health care systems, in part due to fear of deportation. The “UN joint guidance note on equitable access to COVID-19 vaccines for all migrants” calls on States to establish firewalls between health services and immigration authorities to prevent detention or deportation due to accessing health care services [47]. While an important call, limited accountability can further harm those most vulnerable. For example, in Chile, to access COVID-19 vaccines, displaced Venezuelans must self-report to police and disclose their irregular migration status [33]. Policies requiring disclosure of migration status can perpetuate the stigmatization of migrants, particularly as more than 100 migrants, mostly Venezuelans, were deported from Chile in February 2021. Then, in April 2021, another 55 migrants were deported as a part of the “Colchane plan” which sought to deport 1,800 migrants and increase border controls [14]. Similarly, in Peru, irregular migrants must register with the National Registry of Identification and Civil Status, and Brazil requests migrants register with the *Cadastro de Pessoas Físicas* (Naturalized Persons Registry) [43, 48]. Amnesty International has called on States to “explicitly guarantee that vaccination will not be linked to legal status including ensuring that personal data gathered…for vaccination purposes will not be shared with law-enforcement agencies and used for immigration enforcement” [35]. Amid the fears and uncertainties following mass deportations, migrants may avoid registering and declaring their irregular migration status, thus limiting vaccine access.

Meanwhile, in 2021, amid increasing numbers of Venezuelans seeking to return to Venezuela in response to discrimination, xenophobia, and violence in host countries, Venezuelan President Maduro blamed returning migrants for upsurges in COVID-19 cases [49]. Indeed, the Office of the United Nations High Commissioner for Human Rights writes that “in situations of fear and uncertainty, such as the current pandemic, migrants can be particularly vulnerable to attitudes and behaviors that stigmatize and scapegoat them” [47]. Colombian President Duque justified the exclusion of Venezuelans from vaccination campaigns for fear that it would lead to a “stampede” of migrants rushing the border [50]. A senior government official in Venezuela even referred to migrants entering the country illegally as “biological weapons” [51]. Both across country-level policies and related gray literature, our review demonstrates that amid extreme resource constraints facing Latin American countries due to COVID-19 and Venezuelan mass migration, stigmatization and scapegoating can result in discriminatory policies that constrict migrants’ right to the highest attainable standard of health.

In addition to COVID-19 vaccine access specifically, results revealed health care access barriers more broadly for displaced Venezuelans. A report by the Mixed Migration Centre noted that 75 percent of displaced Venezuelans in Peru and 39 percent in Colombia believed they would not be able to access health care if they became ill with COVID-19 [52]. Similarly, compared with citizens, displaced Venezuelans in Chile were 7.5 times less likely to have access to health care [32]. Despite calls from the Pan American Health Organization for “health services [to] be inclusive and responsive to the needs of migrants” and to “[eliminate] geographical, economic, and cultural barriers”, our review revealed that displaced Venezuelans lacking formal documentation were disproportionately affected by health care access barriers [53]. While the integration of displaced Venezuelans into COVID-19 vaccination plans is an important step towards health equity, reviewed literature reinforce the need to address social and structural disparities that limit the right to health for migrants.

## Discussion

These findings advance understandings of migration status as a determinant of COVID-19 vaccine equity by presenting a regional analysis of how country-level COVID-19 vaccination policies in Latin America facilitate access for migrants and displaced people. While the scale and impact of Venezuelan mass migration were underscored in the peer-reviewed and gray literature reviewed, government plans and policies highlighted that States’ provided limited safeguards for non-citizens with precarious or irregular status. Our results underscore the imperative to not only recognize but also address protections for non-citizens in Latin America. International human rights principles reinforce the obligations of States to ensure equity for all in access to COVID-19 vaccines, regardless of citizenship or migration status.

The potential impact of our results not only help underscore how existing gaps in COVID-19 vaccination could have been addressed by human rights approaches, but also advance understandings of how future public health interventions can better integrate human rights principles to complement strategies informed by epidemiological risk. Existing and pervasive disparities highlight how COVID-19 vaccines, no matter how effective at preventing infection or severe illness, cannot reach their full promise without the integration of context-specific sociopolitical factors in their rollout. Human rights-related concerns surrounding COVID-19 vaccine access for displaced Venezuelans include: lack of prioritization of migrants in vaccine distribution; onerous documentation requirements to be eligible for COVID-19 vaccination; and how pervasive anti-migrant discrimination limited equitable health care access. These findings align with reports from the United Nations International Organization for Migration and WHO that country-level vaccination plans often lack clarity regarding the inclusion of migrants and fail to address underlying health care access barriers, particularly for migrants with irregular status [54].

This research builds on existing literature urging the integration of human rights perspectives in responses to COVID-19 to ensure health equity for migrants [54]. We have identified key priorities when designing and implementing COVID-19 vaccination plans to ensure equity for migrants and/or displaced people in the Latin American region. Paralleling literature detailing the urgency of incorporating human rights perspectives into vaccine deployment, planning and coordination mechanisms must account for non-citizens, particularly those with irregular migration status [54]. More broadly, mechanisms need to be adopted to address pervasive discrimination and ensure secure and safe access to public health systems for migrants. Assessment of priority groups within country-level vaccination policies alongside gray and peer-reviewed literature allows for an examination of how governments initially conceptualized access to vaccines for migrants and mobile populations amid a pandemic. Fig 1 situates five of the principles guiding the allocation and prioritization of COVID-19 vaccination as described by the WHO/SAGE framework alongside recommendations for how existing gaps could have been addressed through the integration of human rights.

Recommendations based on this review build on the WHO/SAGE framework which outlines six principles guiding COVID-19 vaccine prioritization, and underscores how social vulnerabilities (including migration status) should inform national vaccination policies. These include: (1) human wellbeing; (2) equal respect; (3) global equity; (4) national equity; (5) reciprocity; and (6) legitimacy. The WHO/SAGE framework calls for migrants to be prioritized in COVID-19 vaccination plans given their increased social vulnerabilities and existing barriers in accessing health services. For example, the principle of “national equity” advocates for the prioritization of vulnerable communities, such as people living in poverty, refugees, and internally displaced persons, those affected by humanitarian emergencies, and irregular migrants. Recognizing their increased risk of COVID-19 acquisition and severe illness, the WHO/SAGE framework underscores why migrants *should* be named as priority groups for COVID-19 vaccination. Yet, as evidenced in our review, scant attention was paid to accountability in implementation of country-level vaccination plans, resulting in States prioritizing the vaccination of citizens over non-citizens.

Fig 1 builds on a modified WHO/SAGE Framework to present how COVID-19 vaccine allocation and prioritization can better consider the health rights of migrants and non-citizens. For example, while the existing WHO/SAGE framework suggests that all people should be treated with equal respect, human rights obligations hold States accountable for ensuring dignity and equal respect for all. Similarly, while the WHO calls for “a transparent consultation process” in decisions surrounding COVID-19 vaccine policies, a rights-based approach necessitates that made-vulnerable communities participate actively in decision-making processes. Finally, extraterritorial obligations underscore the responsibility of States to uphold the health rights of all people, not just those residing within their national borders. Reciprocity is a principle of narrower scope compared to the other five as it specifically addresses those within society “who bear substantial additional risks and burdens of COVID-19 response for the benefit of society” [22]. While many migrants are trained as health care workers and/or first responders, they are often unable to work in these fields given challenges with documentation, and thus do not qualify under this principle. Assessment of policies related to whether migrants and other displaced people can provide COVID-19 care irrelevant of documents was outside of the scope of our review, and thus reciprocity is not presented in Fig 1. Nonetheless, future research is needed to better understand how the principle of reciprocity can be interpreted and applied to migrants and other displaced people, especially given known shortages of healthcare workers in the Latin American region [55].

While the WHO/SAGE framework underscores the need to consider social vulnerabilities, our review revealed that country-level vaccination plans rarely accounted for the structural inequities driving increased exposure to COVID-19 for marginalized communities. These findings support literature evidencing how unstable housing, precarious employment, discrimination, and health care access barriers represent intersectional structural-level factors that constrain access to social protections not only for migrants, but also for other marginalized groups [56]. Prioritizing vaccination based solely on epidemiological risk groups serves to individualize risk and invisibilize the structural-level factors that produce group vulnerabilities. As argued by Sekalala and colleagues, “international human rights law requires that ‘vulnerability’, if used as a criterion for priority access to COVID-19 vaccines, must include social vulnerability (e.g. socioeconomic status) in addition to medical vulnerability (e.g. comorbidities), and attend to intersectionalities” [23]. In this way, prioritization of COVID-19 vaccination must be informed not only by epidemiological risk of morbidity and mortality; a human rights perspective would also require social vulnerabilities be considered when defining vaccine priority groups. As shown by Freier et al., this position has been adopted by the Inter-American Commission on Human Rights and the Inter-American Court of Human Rights, who established that the human rights principles of equality and non-discrimination requires States to not only prohibit discrimination based on migration status, but also to take special measures to address and reverse structural and systemic discrimination, exclusion, and vulnerability faced by migrants [57]. In countries where migrants already faced high rates of discrimination, xenophobia, criminalization, poverty, exploitation, and violence, COVID-19 exacerbated inequities in employment, health, and access to social services [58].

Findings underscore that sustained attention must be paid to the structural inequities described above that are not new, but rather pervasive consequences of neoliberal globalization. This call to center vulnerability seeks to underscore the broader systemic oppressions that make certain communities in Latin America particularly vulnerable to COVID-19 and other epidemics. It demands attention to be paid to imperialist structural adjustment policies that have left public health systems underfunded and ill-equipped to deal simultaneously with mass migration and a pandemic. Indeed, in response to the emerging debt crisis in the 1980s, countries across Latin America accepted loans from the World Bank and International Monetary Fund; conditions of these loans included mandates to privatize state assets, cut government spending, devalue currencies, and liberalize trade [59]. These large-scale cuts to government spending on social services widened health inequities as health care became increasingly unaffordable for the most vulnerable, including migrants [59]. The prioritization of COVID-19 vaccines in Latin America must be situated within ongoing neoliberal reforms yielding chronically underfunded social systems, including health care [60]. In sum, COVID-19 has repeatedly revealed the porousness of borders, yet responses continue to neglect the globalized and interconnected nature of the pandemic.

Caution should be observed in the interpretation of these results as findings are inherently limited by dynamic changes in approaches to COVID-19 vaccination that have occurred since this scoping review was conducted between April and June 2021. Review of country-level vaccination plans revealed at times daily shifts in vaccine prioritization and allocation, as well as migrants’ eligibility for vaccination, underscoring how rights claims were contested, mediated, and resolved across the study period. Indeed, in October of 2021, Colombia extended COVID-19 vaccine access to migrants with irregular status, including those without documentation [61]. While this policy change underscores how our findings must be understood within the context of initial vaccination efforts, there are important and lasting implications of these initial responses which merit further investigation. That said, given the scope of our three-month data collection period, it was not possible to fully assess how COVID-19 vaccination policies shifted across time. Additionally, vaccination data published across Ministry of Health websites did not disaggregate based on migration status, so the effects of COVID-19 vaccination policies on access to vaccines in practice could not be assessed. Further attention is also needed to the phases of vaccination, as well as definition of priority groups across different settings, not only in initial doses, but in the administration of booster doses also. Nonetheless, this review underscores ongoing gaps in COVID-19 vaccination plans as well as the crucial need to uphold international human rights obligations and consider the unique social vulnerabilities of displaced Venezuelans. As Peru and Brazil have done for Indigenous communities, this could be accomplished through an approach to vaccination that considers not just individual-level risk factors but also intersectional, structural-level factors that place displaced Venezuelans at increased risk for COVID-19. The application of a rights-based approach also highlights key tensions surrounding accountability for upholding the health rights for migrants and non-citizens, who can fall through the cracks of social protections amid intersecting crises such as COVID-19 and Venezuelan mass migration. Ensuring equitable access to COVID-19 vaccines is thus a first—and an essential—step to respond to COVID-19 more equitably and prepare for future public health crises in the context of displaced populations.

## Conclusion

Amid rapidly evolving COVID-19 vaccination plans, findings presented here highlight how anti-migrant discrimination can be reinforced by governments and health systems when the human rights of migrants are not accounted for in vaccination policies across Latin America. A human rights approach provides a framework to advance global public health through legally binding international treaties and soft-law materials which transform moral imperatives into legal entitlements [23]. Countries across the region prioritized the allocation of limited vaccines to ‘at-risk’ groups as defined by countries and guided by primarily epidemiological factors (age, occupation, co-morbidities, etc.). Limited attention to social vulnerabilities (such as housing instability, economic precarity) and global forces driving the migration crisis (such as imperialism and neoliberalism) has exacerbated deep-rooted structural inequities faced by those abandoned by social safety nets, such as migrants. Keen attention and deeper inquiry are needed to the unique barriers faced by Venezuelan migrants with irregular status, defined as those who entered a country without the necessary authorization or documentation. Requirements for identification and other government-issued documentation to access vaccines creates critical access barriers (e.g. fear of deportation and xenophobic harassment, lost/expired documents), particularly for migrants with irregular status. Meanwhile, issues of cost, lack of familiarity with health care systems, and stigma and discrimination are intersectional issues that may be more salient among irregular migrants, particularly amid COVID-19 and future pandemics [62]. Stigmatization and scapegoating can result in discriminatory policies that further limit the ability of migrants to achieve the “highest attainable standard of health”.

The integration of human rights, rooted in legally binding treaties that can be used in litigation and advocacy, as well as soft law materials, into public health responses is a key strategy to improve accountability within implementation and uphold the health rights of marginalized and made-vulnerable communities. While this research predominantly analyzed country-level vaccination plans, these are just one component of multi-level vaccination strategies, which often involved provincial, municipal, or local-level governments also. To ensure health equity for migrants and displaced people, future COVID-19 vaccination strategies in Latin America must be informed not just by epidemiological approaches, but also by international human rights.
